# Development of a LAMP-Based Diagnostic for the Detection of Multiple HIV-1 Strains

**DOI:** 10.3390/bios14040157

**Published:** 2024-03-27

**Authors:** Amy Makler-Disatham, Massimo Caputi, Waseem Asghar

**Affiliations:** 1 Micro and Nanotechnology in Medicine, College of Engineering and Computer Science, Florida Atlantic University, Boca Raton, FL 33431, USA; amakler@my.fau.edu; 2 Department of Electrical Engineering and Computer Science, Florida Atlantic University, Boca Raton, FL 33431, USA; 3 College of Medicine, Florida Atlantic University, Boca Raton, FL 33431, USA; mcaputi@health.fau.edu

**Keywords:** HIV-1, LAMP, point-of-care, diagnostics

## Abstract

Since its first appearance in 1981, HIV-1 has remained a global concern. Current methods for diagnosing HIV-1, while effective, are mostly specific to a given subtype of HIV-1 and often require expensive equipment and highly trained individuals to collect and process the sample. It is necessary to develop a sensitive diagnostic method that can be administered with minimal equipment to provide better care in low-resource settings. Loop-mediated isothermal amplification is a rapid and sensitive method for detecting the presence of specific nucleic acid sequences. Herein we report the development and comparison of two different HIV LAMP assays, integrase and VPR, as well as the comparison between TRIZol and magnetic beads RNA extraction methods for each assay. Our analysis shows that the integrase assay was able to detect the virus from multiple subtypes in under 30 min with a variable limit of detection (LOD) that was dependent on the HIV-1 subtype.

## 1. Introduction

Since the Human Immunodeficiency Virus type I (HIV-1) was first described in 1981, more than 85 million people have been infected, and about 40 million have died from acquired immunodeficiency syndrome (AIDS), the terminal result of the HIV-1 infection [1,2]. In 2022, approximately 39 million people worldwide were living with HIV, of which more than 25 million were in sub-Saharan Africa [3,4]. In the United States, there are about 1.2 million people living with HIV [5]. An estimated 32,100 new cases and an additional 36,136 new diagnoses were reported in 2021. It is necessary to increase surveillance and improve the current diagnostics to eradicate the disease [6,7,8].

The initial acute phase of the viral infection lasts approximately 2–5 weeks and is characterized by the uncontrolled replication of the virus, which spreads all over the body and reaches high titers (>2 × 10^7^) [9]. In this phase, most patients manifest flu-like symptoms, such as fever, rash, arthralgia, and headache, and experience a drop in the CD4 T lymphocyte counts caused by the cytopathic effects of the virus. Anti-HIV antibodies are detectable 2–4 weeks after the initial infection. The increase in the anti-HIV antibody titer correlates with the rapid decrease in the circulating virus and the rebound of the CD4+ T lymphocyte population. The increase in the anti-HIV antibody titer and rebound of the CD4+ T cell population correlates with the chronic stage of the HIV infection, which, in the absence of antiretroviral therapy, will progress to AIDS in 5 to 10 years, although the timeframe might vary greatly among individuals [10]. In the chronic stage of the viral infection, the number of CD4+ lymphocytes declines steadily while the virus keeps replicating, although at a lower level than in the acute phase [1,2]. This phase is characterized by a constant decline in the immunocompetent status of the patient and an increased susceptibility to opportunistic infections. Once the CD4+ T cell level drops below a certain threshold, which varies greatly among individuals but is usually between 200 and 500 cells/mm^3^, anti-HIV antibody production is reduced, and viral replication increases. The lower CD4+ T cell count and higher viral titer correlate with a series of bacterial, viral, fungal, and protozoal infections, which together with neurological symptoms characterize the AIDS stage of the viral infection, which without treatment leads to death in 3 years or less [10,11]. Progression to AIDS is now a rare event thanks to highly efficient therapeutics utilized in anti-retroviral therapy (ART) that block viral replication and stop most HIV-related illnesses and infections. In patients receiving ART, the virus enters a latent, non-productive, suppressed infectivity phase that results in levels that are below the sensitivity threshold of current blood tests (>100 copies/mL) [12].

The most commonly used primary screening for HIV is ELISA (enzyme-linked immunosorbent assay), which is used to detect the presence of either, or both, the primary antibodies of an immune response against HIV as well as the viral antigen [10,13,14,15]. However, ELISA rapid tests cannot reliably detect the presence of anti-HIV antibodies before an adequate response is mounted (3–4 weeks) [13,16]. Positive primary tests are then confirmed with enzyme immunoassays (EIA) that detect viral antigens and/or nucleic acid tests (NAT), which enable the detection of extremely low viral loads (50–100 genome copies/mL) [10,17]. Viral load (VL) testing in people living with HIV is required to monitor the efficiency of antiretroviral therapy (ART) and the emergence of drug-resistant mutations throughout the course of the infection. RT-qPCR assays are routinely utilized for VL testing since they are highly sensitive and quantitative; however, they require trained personnel and equipment that is often lacking in point-of-care (POC) centers and low-income/low-resource settings [13,18].

RT-qPCR has long been the standard for NAT analysis for HIV. However, the process of preparing a sample and performing RT-qPCR is laborious and time-intensive, requires expensive equipment like centrifuges, thermocyclers, and qPCR machines, and requires highly trained personnel to perform the RNA extraction and utilize the machines. An alternative to RT-qPCR is loop-mediated isothermal amplification (LAMP). LAMP is a robust technique that can be utilized as an alternative to RT-qPCR and allows the amplification of a target sequence at constant temperature in less than one hour. LAMP’s isothermal properties stem from the three primer pairs used: the forward and backward primers (F3/B3), the inner primers (forward inner primer, backward inner primer; FIP/BIP), and the loop primer pair (forward loop and backward loop; LF/LB). LAMP does not require a thermocycler, leading to a more cost-effective solution that can be utilized in underdeveloped and resource-limited regions [13,19].

In this paper, we report the development of a LAMP assay that targets the integrase (IN) and VPR regions of the HIV-1 genome and is capable of detecting the HIV-1 M-group subtypes A, B, C, D, E, F, and G with high accuracy in 30 min. We also compared TRIzol and Dynabeads RNA extraction methods to determine the potential for a beads-based isolation method in resource-limited settings, as well as preliminary assessment for diagnostic potential with a LAMP-based HIV assay.

## 2. Materials and Method

### 2.1. Cell Transfections

HEK293 cells were transfected with 1.5 mg of the HIV-1 molecular clone pNL4-3 utilizing the Lipofectamine 2000 (Thermo Fisher Scientific, Waltham, FL, USA) transfection reagent according to the manufacturer’s instructions in 100 mm plates. Following the transfection, the cells were incubated for 24 h, washed 2x with PBS, and incubated in fresh DMEM for 48 h before harvesting the supernatant. The supernatant was then centrifuged at 2500 rpm for 5 min to remove debris, divided into 250 μL aliquots, and stored at −80 °C. The cells were then cultured for an additional 24 h before the supernatant was harvested again. After the removal of the supernatant, cells were lysed by adding 6 mL of TRIzol™ to the plate. The TRIzol™–cell mixture was then aliquotted into 1 mL tubes and stored at −80 °C until needed.

### 2.2. RevCEM-D4 Cell Infection

HEK293 cells were transfected with the HIV-1 molecular clone pNL4-3 utilizing Lipofectamine 2000 (Thermofisher) manufacturer instructions. After 72 h, the supernatant was collected and utilized to infect RevCEM-D4 cells (obtained through the NIH HIV Reagent Program, Division of AIDS, NIAID, NIH: ARP-13437, contributed by Dr. Alex Sigal). RevCEM-D4 cells were cultured at 20,000 cells/mL in a 12-well plate with 1 mL of RPMI (no antibiotics) and infected with 100 μL of the pNL4-3-transfected HEK293 supernatant or with 100 μL of mock-transfected control HEK293 supernatant. The RevCEM-D4 cells were incubated for 24 h, washed with PBS, resuspended in fresh RPMI media, and re-plated. The cells were then left to culture for an additional 72 h before collection and storage at −80 °C.

### 2.3. HIV Strains

Plasma samples from HIV-1 patients for multi-strain detection were obtained through the NIH HIV Reagent Program, Division of AIDS, NIAID, NIH. In total, 55 distinct patient isolate samples, encompassing strains A–G, were acquired and utilized to test the LAMP primer sets and were stored at −80 °C until ready for use. The list of distinct HIV-1 samples, their strains/subtypes, and the NIH AIDS Reagent Program catalog numbers are provided in Supplementary Table S1.

### 2.4. RNA Extraction

TRIzol™ (Thermo Fisher Scientific, Waltham, MA, USA) was used for HIV-1 RNA isolation from plasma samples. A total of 750 μL of TRIzol was used to process 250 μL of plasma, which was incubated at room temperature for 5 min. A total of 200 μL of chloroform was added to the solution and vortexed before centrifuging for 5 min at 4 °C at 13.2× *g*. The aqueous phase was captured and mixed with 1 mL of 100% ethanol. A total of 20 mg of glycogen (Thermofisher) was added to the reaction, and the sample was incubated at −80 °C for 30 min. The sample was then centrifuged 13.2× *g* at 4 °C for 20 min to pellet the RNA. The supernatant was removed, and the pellet was washed with 500 μL of 70% ethanol, centrifuged, and resuspended in 50 mL of ddH_2_O. The eluted samples were DNAsed utilizing TURBO DNase (Thermofisher) at 37 °C for 30 min to remove plasmid DNA contaminants. Following the DNAse reaction, 400 mL of ddH_2_O, 50 mL of 3M sodium acetate pH 5.2, and 500 μL of acid:phenol chloroform were added to the sample and centrifuged for 5 min at room temperature. The aqueous phase was collected, mixed with 1 mL of 100% ethanol, and incubated at −80 °C for 30 min. The sample was then centrifuged at 13.2× *g* at 4 °C for 20 min, the supernatant was removed, and the pellet was washed with 500 μL of 70% ethanol, centrifuged for 5 min at 4 °C, and left to dry for 5–10 min at room temperature before resuspending it in 50 μL of ddH_2_O.

The Dynabeads™ SILANE Viral NA Kit (Thermofisher) was also utilized for the extraction of viral RNA from culture supernatants and plasma following the manufacturer’s instructions, except for the RNA being eluted in 50 μL of Invitrogen™ UltraPure™ DNase/RNase-free distilled water.

For both TRIzol and beads extraction, 200 μL of plasma was spiked with 50 μL of viral sample.

### 2.5. LAMP Primer Design and Testing

Multiple HIV-1 strains were aligned using the HIV Los Alamos National Library HIV Sequence Database (http://www.hiv.lanl.gov/ ( accessed on 15 February 2024). Visualization of the alignments was conducted using the Portugene HIVOligoDB and Wasabi databases [20] and Geneious Prime 2023.2.1 (https:www.geneious.com, http://www.hiv.lanl.gov/ (accessed on 15 February 2024)). LAMP primers were designed against HIV-1 pol-integrase(IN)-Vif region (GenBank Accession# AF033819.3, nucleotide positions 4437–4620) and VPR (GenBank Accession# AF033819.3, nucleotide positions 5190–5430) using the LAMP primer design tool from Primer Explorer V5 from Eiken Chemical Co. Ltd. (Tokyo, Japan). The target sequences are reported in Supplementary Table S2, and the primer sequences are reported in Supplementary Tables S3 and S4.

The HIV-1 NL4-3 virus was used for initial testing and validation of the LAMP primer sets. A LavaLamp™ RNA MasterMix Kit (Lucigen Corporation, Middleton, WI, USA) was used for all RT-LAMP reactions. The LavaLamp™ RNA MasterMix reaction was prepared as per the manufacturer’s instructions. The AriaMX Real-Time PCR system (Agilent Technologies, Santa Clara, CA, USA) was utilized to maintain a temperature of 70 °C for 40 min in all the experiments.

### 2.6. Quantification of the Limit of Detection

The limit of detection of the LAMP assay for each HIV subtype was determined using viral clones obtained through the NIH HIV Reagent Program, Division of AIDS, NIAID, NIH: ARP-12637, pQ23x (Q461.e2), subtype A; ARP-3443, subtype B; ARP-6439, pMJ4, subtype C; ARP-4002, p94UG114.1.6, subtype D; and ARP-4005, p90CF402, subtype E. Target sequences were generated for subtypes F and G using the integrase sequences of BZ163 (AY173958.1) and HH8793 (AY214079.1), respectively (Integrated DNA Technologies).

All samples were serially diluted 1:3 to produce a standard qPCR curve before assessing the limit of detection (LOD) for the LAMP assay across tested subtypes. For all LAMP reactions, 5 μL of the sample was used and run in technical duplicates. The LOD was determined once no more signal was produced in the Agilent AriaMX Real-Time PCR system.

## 3. Results

**Figure 1:**
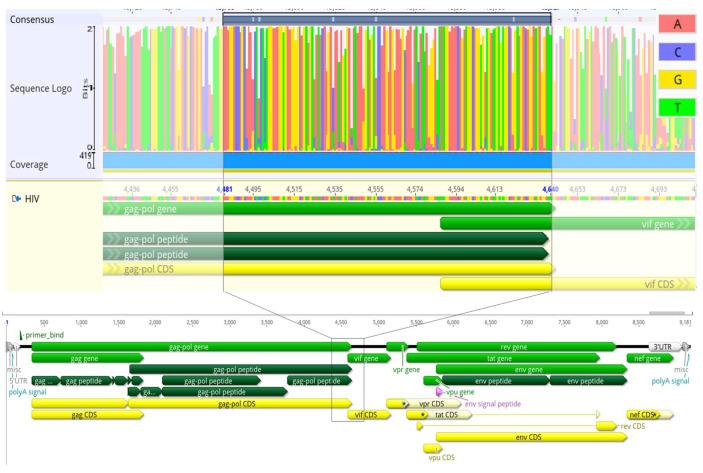
Alignment of the *pol*-integrase of HIV-1 isolates from 422 sequences across all M group subtypes and 19,628 B subtype sequences for VPR gene. The alignment was conducted using HIV Los Alamos (https://www.hiv.lanl.gov/ (accessed on 15 February 2024)) and Geneious Prime 2023.2.1 (http://www.geneious.com/ (accessed on 15 February 2024)) [20,21]. HIV-1 *pol*-integrase alignment utilized for the development of IN-specific LAMP primers; relative reference HIV HXB2, nucleotide position 3770–4640. Red—A, blue—C, yellow—G, and blue—T.

To develop an HIV-1 multi-strain LAMP-based diagnostic assay, we first conducted a multiple alignment of HIV-1 subtypes utilizing the Los Alamos HIV Database (http://www.hiv.lanl.gov/ (accessed on 15 February 2024)) and then visualized the alignments using HIVOligoDB [20] and Wasabi (Figure 1) [21]. These alignments aided in visualizing conserved regions with a length of ≥200 nucleotides for LAMP primer design. Through this assessment, the portion of the *Pol* gene that encodes for the Integrase protein was identified as an ideal region across all HIV-1 subtypes for developing LAMP primers. Four distinct LAMP primer sets were designed using the primerexplorer.jp LAMP primer V5 design tool (Supplementary Table S3).

### 3.1. Validation of LAMP Integrase Primer Sets

**Figure 2:**
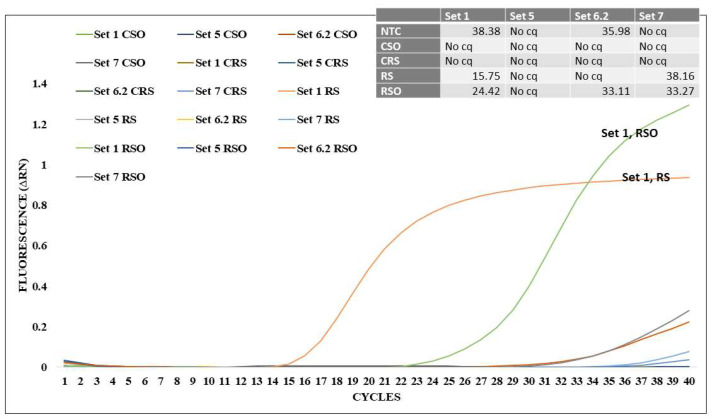
LAMP primer set analysis against the integrase region. Four LAMP primer sets were generated against the integrase-coding region, named in this figure as Set 1, Set 5, Set 6.2, and Set 7. HEK293 cells were transfected with HIV-1 NL4-3 isolate. The supernatant of the transfected HEK293 cells was used to infect RevCEM-D4 cell cultures. The primer sets were tested against cells and supernatant, or supernatant only from RevCEM-D4 D4 cells. NTC—No template control; CSO—Control RevCEM-D4 cells, supernatant only; CRS—Control RevCEM-D4 supernatant and cells; RS—RevCEM-D4 supernatant and cells; RSO—RevCEM-D4 supernatant only.

The LAMP primer sets were tested against RevCEM D4 leukocytes infected with HIV viral clone NL4-3 and/or the purified culture supernatant, as well as cultures that were not infected. Primer set 1 (IN-1) was the most sensitive, providing a time to result (TTR) of 15 min compared to 30 min for primer sets IN-6.2 and IN-7 (Figure 2).

LAMP primer sets require two different pairs of primers and an optional third pair of loop primers: forward (F3)/backward (B3), front inner primer (FIP)/backward inner primer (BIP), and forward loop (LF) and backward loop (LB). These three pairs can be mixed in varying ratios to increase sensitivity and TTR. We therefore analyzed which primer ratios offered the greatest sensitivity (Supplementary Figure S1). The LAMP primers were mixed in different ratios: 1:4:2, 1:8:4, and 1:10:5 of the F3/B3:FIP/BIP:LF/LB. Through this analysis, the optimal ratio of F3/B3:FIP/BIP:LF/LB was determined to be 1:8:4. 

The four LAMP primer sets designed for the *pol-integrase* region were tested against RevCEM D4 leukocytes infected with HIV viral clone NL4-3 and/or the purified culture supernatant as well as cultures that were not infected. Integrase primer set 1 (IN-1) was the most sensitive, providing a time to result (TTR) of 15 min compared to 30 min for primer sets IN-6.2 and IN-7 (Figure 2).

### 3.2. IN-1 LAMP Primers Can Be Utilized to Amplify Several HIV-1 Subtypes

To determine the LOD for the IN-1 primer sets, we utilized DNA plasmid molecular clones coding for the HIV-1 A, B, C, D, and E subtypes. For the F and G subtypes, the integrase sequences were synthesized. Serial dilutions were performed starting with approximately 1 × 10^7^ copies/mL to 1 copy/mL, with the exception being for subtype B, which ranged from 1.5 × 10^5^ copies/mL to 1 copy/mL.

**Figure 3:**
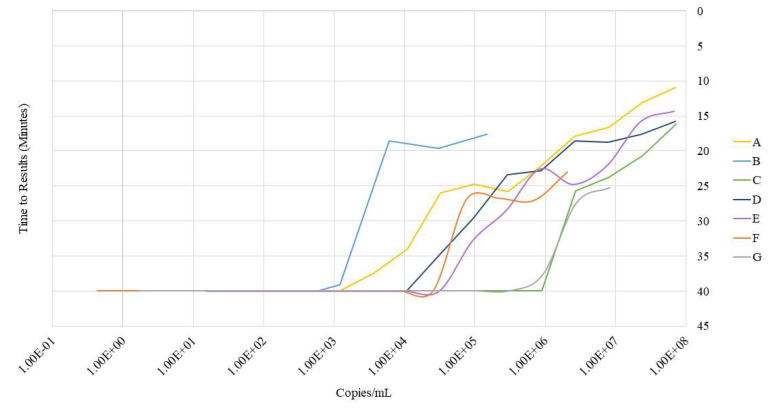
Limit of detection for the IN-1 LAMP primer set across subtypes. Plasmid vectors as well as known viral quantitative standards were used to determine the limit of detection of the IN-1 LAMP primer set across different HIV-1 subtypes.

The IN-1 primers exhibited a LOD from 6 to 1 × 10^4^ copies/reaction. The worst LOD was for subtype C, with a LOD of 1.30 × 10^4^ copies/reaction, while the primers were most sensitive to subtypes A and B, with LODs of 18 and 6 copies, respectively. The primers obtained a LOD of 160 copies/reaction for D, 450 copies/reaction for E, 1.25 × 10^3^ copies/reaction for F, and 4.50 × 10^3^ copies/reaction (Figure 3).

### 3.3. Detection of HIV-1 from a Panel of HIV Isolates

To validate the reliability of IN-1 for multiple HIV-1 subtype detection, 55 HIV-1 cultured isolates of subtypes A-G were acquired from the NIH HIV Reagent Program/BEI Resources (Supplementary Table S1). 

**Figure 4:**
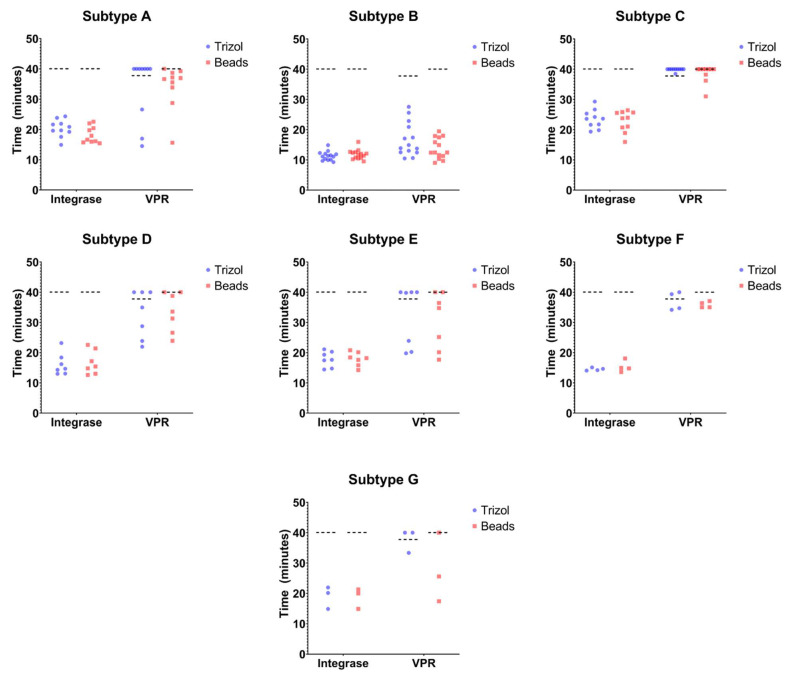
Analysis of viral isolates from subtypes A-G. RNA was extracted from cell-free viral preparations of viral isolates from 7 viral subtypes (A to G). Two RNA extraction methods were compared (TRIzol and magnetic beads for IN and VPR across HIV-1 subtypes A–G). TRIzol or magnetic beads were used to extract HIV-1 RNA from plasma samples containing HIV-1 subtypes A–G. The extraction methods were assessed through LAMP using primer sets designed against integrase, or VPR. The time, in minutes, is provided for each condition. The cutoff time was set to 40 min.

IN-1 primer sets were able to accurately detect the presence of all seven subtypes of HIV-1 (A–G) within 30 min. The B subtype exhibited the shortest average TTR of about 11.5 min, while subtype C showed the longest average TTR of about 24 min. The average of the remaining subtypes fell between these time points (Figure 4). 

To better understand the plausibility of this assay for field use in resource-limited regions, beads-based RNA extraction was compared to standard TRIzol extraction. Beads-based RNA extraction is efficient and does not require expensive and bulky equipment or potentially hazardous reagents, whereas standard TRIzol extraction does. Therefore, beads-based RNA extraction offers greater field versatility. Its efficiency was examined against TRIzol for IN-1 and all tested subtypes. The data show that a consistent TTR was observed for each sample, regardless of the extraction method (Figure 4).

We also tested additional primer sets that we hypothesized to be sub-optimal due to less conservation across subtypes. When we assessed these additional primer sets designed to target the VPR region against the 55 isolates, we observed that the TTR ranged from 10 to 40 min with little consistency within replicates. Consistency was observed, however, when comparing the RNA extraction methods. The most reliable TTR for VPR-2 was observed in subtype B, with a TTR under 30 min for TRIzol and about 20 min for beads-based extraction.

The comparison of IN-1 to VPR-2 primers and TRIzol to beads-based RNA isolation showed greater consistency with the IN-1LAMP primer set, regardless of the isolation method. By comparison, the VPR-2 LAMP primer set showed a higher degree of variability between the two different isolation methods as well as within the technical replicates. The IN-1 assay was also able to detect all HIV-1 subtypes that were tested, while the VPR-2 primer set was only able to reliably detect subtype B. The IN-1 primer set did not produce a signal in the HIV-free plasma control sample in either isolation method, whereas VPR-2 showed a signal in the TRIzol-extracted sample after 37 min. IN-1, therefore, seems to be a more reliable primer set compared to VPR-2. 

## 4. Discussion

HIV RT has a high mutation rate of 1 in 10,000 nucleotides per replication cycle due to an inability to proofread. If the virus is left to replicate without interference, it can generate nearly a billion viral particles per day with mutations that will be incorporated into the host genome. This rapid mutation rate contributes to evading the immune response, high genetic variation within a subtype (15–20%), and difficulty treating patients effectively [10,22,23]. Thus, the sensitivity and efficiency of NATs can vary between subtypes and even within the same subtype.

HIV is a pandemic and public health issue that disproportionately affects people in underdeveloped countries, though recent years have seen a steady decline in the number of cases reported per year. However, from 2010 to 2019, there was an observed increase in cases within the USA, with around 1.2 million cases as of 2020, with subtype B being the most common [2,24]. Regardless, the number of deaths has decreased thanks to the increased use of antiretroviral therapies, which have helped patients maintain undetectable levels of the virus and achieve a normal lifespan. It is therefore imperative that patients are able to learn of their diagnosis as soon as possible, as this could promote faster adoption of ARTs and therefore decrease the spread of HIV.

A rapid diagnostic that can be disseminated to underdeveloped countries and point-of-care settings can facilitate HIV detection, mitigate spread, and promote the use of ART sooner to halt disease progression. There have been assays developed previously for HIV utilizing LAMP [25,26,27]. Ocwieja et al. reported that their RT-LAMP assay was able to detect subtypes A, B, C, D, and G with a LOD of 5000 copies within 30 min [27]. Another RT-LAMP HIV-1 multi-strain detection study exhibited a LOD of 1200 copies/mL for HIV-1 group M subtypes [26]. A third study developed a silicon microfluidic chip with cell-phone imaging to detect LAMP amplicons [25]. This assay was able to detect about 60 viruses in a 1 nL sample of whole blood, which they report as corresponding to 670 viruses/μL. More recently, a multi-strain detection assay was developed that was able to detect all M subtypes, including recombinant forms, with the ability to detect around 10,000 copies/mL [28]. Interestingly, similar to our study, these previous studies all utilized the IN-coding region. Additionally, these assays appear to exhibit a range of sensitivity from 1200 to 10,000 copies/mL, of which our assay either shows superiority or comparability.

In this study, we compared the efficacy of IN to VPR and found that VPR exhibited greater variability and decreased reliability in its ability to detect multiple HIV-1 subtypes, with its more consistent detection being observed with subtype B isolates. Two different RNA extraction methods were also compared: TRIzol and magnetic beads-based isolation. This study found that, for IN, there was no appreciable difference between the extraction methods. VPR, on the other hand, was unreliable in its detection of multiple strains, regardless of the extraction method. 

Our study identified a reliable and consistent multi-strain LAMP-based assay that targeted the integrase-coding region of HIV-1. This LAMP-based detection method exhibited a LOD of 6 copies/reaction for subtype B at an optimum efficiency of 1:8:4 ratio for the F3/B3:FIP/BIP:LF/LB primers. For subtype C in both plasma samples and plasmids, the primer efficiency was low. When the integrase sequence for the primers was compared to the pMJ4 (subtype C) sequence, only 60% similarity was observed. This likely explains why our LOD for C is approximately 1.30 × 10^4^ copies/reaction. Additionally, two different RNA extraction methods (TRIzol and magnetic beads) were compared and found to provide similar efficiency. The variability in detection efficiency of the IN-1 depended on subtype. Our data show that IN-1 is most sensitive to detecting subtype B, being able to achieve so in under 20 min, while for others, the time to results can be as long as 30 min or more. While our assay is able to detect multiple subtypes, it cannot distinguish between subtypes and therefore would require qPCR validation to determine which subtype was detected. These primer sets were not tested against HIV-2 or recombinants, and so we cannot rule out whether the IN primer may have an even broader application than examined in this study. 

Future studies will test the LAMP assay on a microfluidic chip that has previously been used to detect HCV [29] and Zika [30] viruses, therefore allowing us to determine the efficacy of this diagnostic in resource-limited regions of the world. A rapid multi-strain HIV test that requires minimal processing of the sample ahead of time can help minimize cost, and given the low LODs for certain strains and the small sample requirement, there is potential to maximize early detection, which would enable sooner treatment.

## Data Availability

Data is available upon request.

## Supplementary Materials

The following supporting information can be downloaded at: https://www.mdpi.com/article/10.3390/bios14040157/s1, Figure S1: Amplification results for LAMP primers against Vpr region. Three different ratios of the primer sets for Vpr and two for pol-integrase (IN-1 LAMP primers) were tested against three dif-ferent dilutions (copies/ml) of NL4-3. Time to results in minutes is also provided; Table S1: HIV-1 strains and subtypes acquired from the NIH AIDS Reagent Program; Table S2: HIV-1 sequence used for generation of LAMP primer sets; Table S3: LAMP sets generated against the pol-IN-vpu regions; Table S4: Vpr LAMP primer sets.
