# Supplementary material for: Development of a LAMP-Based Diagnostic for the Detection of Multiple HIV-1 Strains
Source: Biosensors (Basel). 2024 Mar 27;14(4):157. doi: 10.3390/bios14040157 (PMC11048192; doi:10.3390/bios14040157)
Supplement: Supplementary file 1 [file biosensors-14-00157-s001.zip › biosensors-2907256-supplementary.pdf]

Supporting Information

Development of a LAMP-Based Diagnostic for the Detection of Multiple HIV-1 Strains

Amy Makler-Disatham [1,2], Massimo Caputi [3] and Waseem Asghar [1,2,*]

[1] Micro and Nanotechnology in Medicine, College of Engineering and Computer Science, Florida Atlantic University, Boca Raton, FL 33431, USA; amakler@my.fau.edu
[2] Department of Electrical Engineering and Computer Science, Florida Atlantic University, Boca Raton, FL 33431, USA
[3] College of Medicine, Florida Atlantic University, Boca Raton, FL 33431, USA; mcaputi@health.fau.edu
* Correspondence: wasghar@fau.edu

Table S1. HIV-1 strains and subtypes acquired from the NIH AIDS Reagent Program.

Catalog#	Name	Subtype
ARP-1650	Human Immunodeficiency Virus-1 92/UG/029	A
ARP-2176	Human Immunodeficiency Virus Type 1 strain 93/RW/018	A
ARP-2178	Human Immunodeficiency Virus Type 1 strain 93/RW/020	A
ARP-11243	Human Immunodeficiency Virus-1 KER2018	A
ARP-11245	Human Immunodeficiency Virus Type 1 KNH1135	A
ARP-11247	Human Immunodeficiency Virus Type 1 KNH1207	A
ARP-11249	Human Immunodeficiency Virus Type 1	A
ARP-11244	Human Immunodeficiency Virus Type 1 KNH1088	A
ARP-2271	Human Immunodeficiency Virus Type 1 strain 93/RW/034	A
ARP-2203	Human Immunodeficiency Virus Type 1 strain 93/RW/029	A
ARP-1722	Human Immunodeficiency Virus-1 92/US/660	B
ARP-1751	Human Immunodeficiency Virus-1 92/BR/003	B
ARP-317	Human Immunodeficiency Virus-1 MN	B
ARP-416	Human Immunodeficiency Virus-1 ADA	B
ARP-629	Human Immunodeficiency Virus-1 A018A	B
ARP-510	Human Immunodeficiency Virus Type 1 (HIV-1) Ba-L	B
ARP-2258	Human Immunodeficiency Virus Type 1 strain QZ4589	B
ARP-10454	Human Immunodeficiency Virus Type 1 strain TYBE	B
ARP-7691	Human Immunodeficiency Virus Type 1 90/TH/BK132	B
ARP-7689	Human Immunodeficiency Virus Type 1 91/US/4	B
ARP-11252	Human Immunodeficiency Virus Type 1 NP1538	B
ARP-11250	Human Immunodeficiency Virus Type 1 94US_33931N	B
ARP-11251	Human Immunodeficiency Virus Type 1 873 90US_873	B
ARP-7692	Human Immunodeficiency Virus Type 1 89/BZ/167	B
ARP-1777	Human Immunodeficiency Virus-1 92/BR/025	C
ARP-2900	Human Immunodeficiency Virus Type 1 strain 93/IN/101	C
ARP-2903	Human Immunodeficiency Virus Type 1 strain 94/KE/105	C
ARP-10051	Human Immunodeficiency Virus Type 1 strain 1176 MB	C
ARP-10056	Human Immunodeficiency Virus Type 1 strain J38 Ma	C
ARP-10052	Human Immunodeficiency Virus Type 1 strain 985 C12M	C
ARP-10047	Human Immunodeficiency Virus Type 1 strain 1165 MB	C
ARP-11253	Human Immunodeficiency Virus Type 1 98US_MSC5016	C
ARP-11255	Human Immunodeficiency Virus Type 1 TZA68/125A	C
ARP-11256	Human Immunodeficiency Virus Type 1 TZA246	C
ARP-1647	Human Immunodeficiency Virus-1 92/UG/001	D
ARP-1952	Human Immunodeficiency Virus-1 93/UG/065	D
ARP-2208	Human Immunodeficiency Virus Type 1 strain 93/UG/086	D
ARP-2305	Human Immunodeficiency Virus Type 1 strain 94/UG/105	D
ARP-1649	Human Immunodeficiency Virus-1 92/UG/024	D
ARP-12375	Human Immunodeficiency Virus 1, MiMa	D

ARP-12374	Human Immunodeficiency Virus 1, FaKi	D
ARP-2167	Human Immunodeficiency Virus Type 1 strain 93/TH/054	E
ARP-2101	Human Immunodeficiency Virus Type 1 strain 91/US/054	E
ARP-2165	Human Immunodeficiency Virus Type 1 strain 93/TH/051	E
ARP-1687	Human Immunodeficiency Virus-1 92/TH/019	E
ARP-2340	Human Immunodeficiency Virus-1 93/TH/072	E
ARP-2392	Human Immunodeficiency Virus-1 93/TH/078	E
ARP-3023	Human Immunodeficiency Virus Type 1 strain CMU02	E
ARP-7709	Human Immunodeficiency Virus Type 1 89/BR/126 (GS 030)	F
ARP-7710	Human Immunodeficiency Virus Type 1 BZ162 (GS 031)	F
ARP-7711	Human Immunodeficiency Virus Type 1 BZ163 (GS 032)	F
ARP-2329	Human Immunodeficiency Virus Type 1 strain 93/BR/020	F
ARP-3509	Human Immunodeficiency Virus Type 1 strain RU132	G
ARP-3508	Human Immunodeficiency Virus Type 1 strain RU570	G
ARP-7712	Human Immunodeficiency Virus Type 1 HH8793	G

Table S2. HIV-1 sequence used for generation of LAMP primer sets.

**Integrase**	atcaggaaagtactatttttagatggaatagataaggcccaagatgaacatgagaaatatcacagtaattggagagcaatggctagtgattttaacctgccacctg<br>tagtagcaaaagaaatagtagccagctgtgataaatgtcagctaaaaggagaagccatgcatggacaagtagactgtagtccaggaatatggcaactagattgt<br>acacatttagaaggaaaagttatcctggtagcagttcatgtagccagtggatatatagaagcagaagttattccagcagaaacagggcaggaaacagcatatttt<br>cttttaaaattagcaggaagatggccagtaaaaacaatacatactgacaatggcagcaatttcaccggtgctacggttagggccgcctgttggtgggcgggaat<br>caagcaggaatttggaattccctacaatccccaaagtcaaggagtagtagaatctatgaataaagaattaaagaaaattataggacaggtaagagatcaggctg<br>aacatcttaagacagcagtacaaatggcagtattcatccacaattttaaaagaaaaggggggattggggggtacagtgcaggggaaagaatagtagacataat<br>agcaacagacatacaaactaaagaattacaaaaacaaattacaaaaattcaaaattttcgggtttattacagggacagcagaaatccactttggaaaggaccagc<br>aaagctcctctggaaaggtgaaggggcagtagtaatacaagataatagtgacataaaagtagtgccaagaagaaaagcaaagatcattagggattatggaaaa<br>cagatggcaggtgatgattgtgtggcaagtagacaggatgaggatt
**Vpr**	aagctgttagacattttcctaggatttggctccatggcttagggcaacatatctatgaaacttatggggatacttgggcaggagtggaagccataataagaattctg<br>caacaactgctgtttatccattttcagaattgggtgtcgacatagcagaataggcgttactcgacagaggagagcaagaaatggagccagtagatcctagactag<br>agccctggaagcatccaggaagtcagcct

Table S3. LAMP sets generated against the *pol*-IN-vpu regions.

**Set 1**	
F3	AAAATTTTCGGGTTTATTACAGG
B3	GCCACACAATCATCACCT
FIP	ATTACTACTGCCCCTTCACCTTAGAAATCCACTTTGGAAAGGAC
BIP	AGTGACATAAAAGTAGTGCCAAGAAGCCATCTGTTTTCCATAATCCCT
LF	CCAGAGGAGCTTTGCTG
**Set 5**	
F3	CAGAAGTTATTCCAGCAGAA
B3	GATTCTACTACTCCTTGAC
FIP	GCTGCCATTGTCAGTATGTATTCAGGGCAGGAAACAGCATAT
BIP	AATTTCACCGGTGCTACGGTTATTTGGGGATTGTAGGGAA
LF	TACTGGCCATCTTCCTGCTAA
LB	TTGGTGGGCGGGAATCAAGC
**Set 6.2**	
F3	GAAGTTATTCCAGCAGAAAC
B3	GATTCTACTACTCCTTGAC
FIP	GCTGCCATTGTCAGTATGTATTAGGGCAGGAAACAGCATAT
BIP	AATTTCACCGGTGCTACGGTTATTTGGGGATTGTAGGGAA
LF	ACTGGCCATCTTCCT
LB	GGAATCAAGCAGGAATT

**Set 7**	
F3	GCAGGAAGATGGCCAGTA
B3	ACTGCTGTCTTAAGATGTTCAG
FIP	ATTCCTGCTTGATTCCCGCCGACAATGGCAGCAATTTCACC
BIP	TTCCCTACAATCCCCAAAGTCAAGCCTGATCTCTTACCTGTCCTA
LF	CGGCCCTAACCGTAGCA

Table S4. Vpr LAMP primer sets.

F3 Set 1	TTTATCCATTTCAGAATTGGGT
B3 Set 1	GCTTTTGTCATGAAACAAACT
FIP Set 1	TCTAGGATCTACTGGCTCCATTTC-GACATAGCAGAATAGGCG
BIP Set 1	AAGCATCCAGGAAGTCAGCC-GGCAATGAAAGCAACACTT
LF Set 1	TGCTCTCCTCTGTCGAGT
F3 Set 2	AAGCTGTTAGACATTTTCCTAG
B3 Set 2	AGGCTGACTTCCTGGATG
FIP Set 2	GGATAAACAGCAGTTGTTGCAGAAT-GACAACATATCTATGAAACTTACG
BIP Set 2	TGGGTGTCGACATAGCAGAATAG-CCAGGGCTCTAGTCTAGG
LF Set 2	CCACTCCTGCCCAAGTATC
LB Set 2	AGAGCAAGAAATGGAGCCAGTAGA

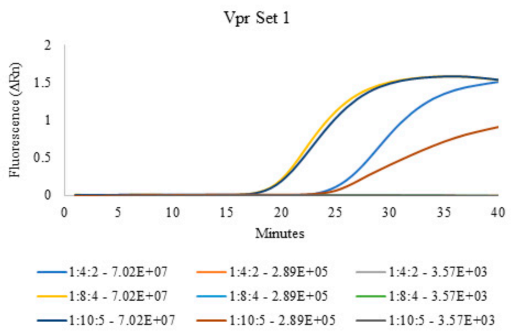

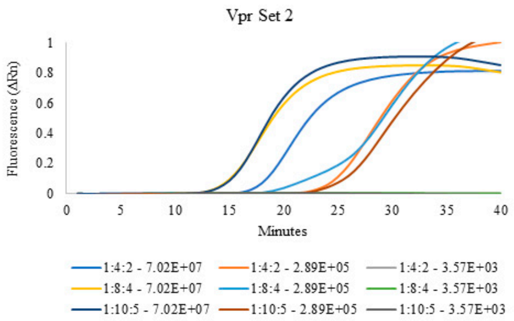

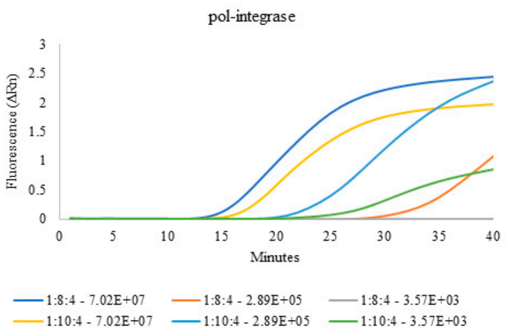

	Primer Ratios	7.02E+07 copies/ml	2.89E+05 copies/ml	3.57E+03 copies/ml
		Time to Results (Minutes)		
Vpr Set 1	1:4:2	23.95	No signal	No signal
	1:8:4	18.33	No signal	No signal
	1:10:5	18.55	26.74	No signal
Vpr Set 2	1:4:2	18.30	24.67	No signal
	1:8:4	14.90	21.94	No signal
	1:10:5	15.04	25.38	No signal
Pol-integrase	1:8:4	14.63	30.74	No signal
	1:10:5	16.37	21.66	25.72

Figure S1. Amplification results for LAMP primers against Vpr region. Three different ratios of the primer sets for Vpr and two for *pol-integrase* (IN-1 LAMP primers) were tested against three different dilutions (copies/ml) of NL4-3. Time to results in minutes is also provided.
